# Beyond the Cornea: Early Changes in Scleral, Iris, and Corneal Parameters After Corneal Collagen Cross-Linking

**DOI:** 10.3390/jcm15124428

**Published:** 2026-06-08

**Authors:** Tunahan Akyol, Osman Parca, Emine Seker Un, Ibrahim Toprak, Gokhan Pekel

**Affiliations:** 1 Department of Ophthalmology, Faculty of Medicine, Pamukkale University, Denizli 20160, Turkey; 2 Department of Ophthalmology, Egekent Hospital, Denizli 20160, Turkey; 3 Department of Ophthalmology, Dünyagöz Hospital, Istanbul 34000, Turkey

**Keywords:** keratoconus, corneal collagen cross-linking, anterior segment optical coherence tomography, scleral thickness, iris thickness, corneal layers

## Abstract

**Background/Objectives:** To evaluate early postoperative changes in scleral and iris thicknesses together with corneal layer thicknesses and tomographic parameters following corneal collagen cross-linking (CXL) in eyes with progressive keratoconus. **Methods:** This retrospective study included 94 eyes of 94 patients with progressive keratoconus who underwent standard epithelium-off CXL using the Dresden protocol. Corneal tomography (Pentacam) and anterior segment optical coherence tomography (AS-OCT) measurements were obtained preoperatively and at the early postoperative follow-up (3 months ± 2 weeks). Thickness measurements of the tear film, corneal epithelium, Bowman layer, stroma, Descemet–endothelium complex, sclera (1–3 mm from the limbus), and iris (1–2 mm from the pupillary margin) were analyzed. Pre- and post-CXL values were compared using paired statistical tests, and effect sizes were calculated. **Results:** In the early postoperative period, scleral thickness showed a significant increase at all measured distances from the limbus, with medium effect sizes, while iris thickness demonstrated a significant decrease at all measurement points with large effect sizes (*p* < 0.001). Tear film, epithelial, and stromal thicknesses decreased significantly after CXL, whereas Bowman layer and Descemet–endothelium complex thicknesses remained unchanged. Pachymetric measurements revealed significant thinning at the pupil center, corneal apex, and thinnest point. No significant changes were observed in Kmax or anterior chamber depth, indicating stabilization rather than progression in the early postoperative period. **Conclusions:** Corneal collagen cross-linking was associated with measurable early structural changes in corneal layers and extra-corneal anterior segment tissues during the postoperative period. The observed increase in scleral thickness and decrease in iris thickness suggest that structural alterations may occur in extra-corneal anterior segment tissues following CXL. These findings support the concept that CXL influences anterior segment biomechanics in a tissue-specific manner and that extra-corneal parameters may serve as complementary markers for early postoperative assessment.

## 1. Introduction

Keratoconus is an ectatic corneal disorder defined by progressive biomechanical weakening of the corneal stroma. It is characterized by stromal thinning and irregular protrusion. The disease usually presents in both eyes, but often not equally. Keratoconus results in irregular astigmatism and worsening visual quality. In progressive cases, it may cause severe visual impairment [1,2]. Because of its progressive nature, halting progression is vital in clinical management.

Corneal collagen cross-linking (CXL) was developed as a therapeutic technique to increase corneal biomechanical strength. It acts by inducing new covalent bonds within stromal collagen fibers. The process uses riboflavin and ultraviolet-A (UVA) irradiation. Currently, CXL is widely accepted as the standard treatment for halting or slowing the progression of progressive keratoconus [3,4]. In addition to the conventional Dresden protocol, accelerated and transepithelial techniques have emerged. The early and long-term effects of these protocols on corneal structure have been reported to vary [5,6].

In clinical practice, the therapeutic response following CXL is primarily monitored using corneal topographic and tomographic parameters, with keratometric values, Kmax, elevation indices, and pachymetric measurements being the most commonly employed metrics. The literature indicates that in the early postoperative period, stabilization or fluctuation, rather than marked keratometric improvement, is usually observed, whereas transient corneal thinning is a frequent finding [7,8,9]. These early changes have been attributed to stromal remodeling, alterations in corneal hydration, and postoperative haze formation [10]. Consequently, interest has increased in more detailed assessments of the structural response to CXL beyond global corneal parameters. In recent years, the importance of evaluating individual corneal layers following CXL has been increasingly emphasized. Analyses of epithelial thickness mapping have demonstrated epithelial remodeling after CXL [3,4], whereas data on early thickness changes in the Bowman layer, stroma, and Descemet–endothelial complex remain limited and inconsistent in the literature.

The present study intends to evaluate, in the early postoperative period after CXL for progressive keratoconus, scleral and iris thicknesses, corneal layer thicknesses, tear film parameters, and corneal topographic and tomographic measurements.

## 2. Materials and Methods

### 2.1. Study Design and Patient Selection

This retrospective study reviewed records of patients with progressive keratoconus who underwent CXL treatment between 2015 and 2024. Ethical approval was obtained before retrospective data extraction and analysis were initiated in accordance with institutional regulations, at the Department of Ophthalmology, Pamukkale University Faculty of Medicine. To avoid inter-eye dependence, only one eye per patient was included. If both eyes met criteria, the more severely affected eye, as shown by tomographic findings, was selected. A total of 94 eyes were included. The study received ethics approval from the Pamukkale University Non-Interventional Clinical Research Ethics Committee (29 July 2025; E-60116787-020-733813) and followed the Declaration of Helsinki.

Doctors diagnosed keratoconus based on eye exams and tests that produce 3D images of the cornea. The condition was considered worsening if follow-up tests showed any of the following: the cornea became more curved, the thinnest area became thinner, or certain test results declined.

Patients were included if they were 18 or older, had progressive keratoconus, and had corneal mapping and Anterior segment imaging was performed with a spectral-domain AS-OCT device (Spectralis OCT, Heidelberg Engineering, Heidelberg, Germany) before and 3 months (±2 weeks) after CXL. Patients were excluded if they had prior corneal surgery (such as a corneal transplant or ring placement), active eye infection or inflammation, corneal scarring or spots, severe eye surface disease that might affect testing, or incomplete or unreliable scans.

### 2.2. CXL Procedure

All patients received standard epithelium-off CXL following the Dresden protocol. After applying topical anesthesia, the corneal epithelium was mechanically removed over an 8–9 mm central zone. Riboflavin 0.1% was instilled every 2–3 min for 30 min. After confirming riboflavin stromal saturation, the cornea was irradiated with UVA at 370 nm and 3 mW/cm^2^ for 30 min. Riboflavin administration continued during UVA irradiation.

At the end of the procedure, each patient received a bandage contact lens. Topical antibiotic and preservative-free artificial tear therapy was started until re-epithelialization. After epithelial healing, topical corticosteroids were introduced and tapered gradually.

### 2.3. Clinical and Imaging Assessments

All patients had a comprehensive ophthalmologic examination before CXL and at 3 months (±2 weeks) after the procedure. The evaluation included corneal tomography (Pentacam HR- Oculus Optikgeräte GmbH, Wetzlar, Germany) and AS-OCT. The same experienced examiner performed all measurements to limit interobserver variability.

Anterior segment imaging was performed with a spectral-domain AS-OCT device with approximately 7 µm axial and 14 µm transverse resolution. Cross-sectional scans were performed using a standardized protocol, with a consistent internal fixation target for alignment. Thickness measurements were made using the device’s built-in caliper tool, with the calipers placed perpendicular to clear anatomical interfaces. Scleral thickness was the distance between the outer scleral boundary (episcleral interface) and the inner scleral border next to the ciliary body. Iris thickness was the distance between the anterior and posterior borders of the iris stroma at set distances from the pupillary margin. Only high-quality images with clear anatomical boundaries were included for measurement reliability.

Using corneal imaging, doctors recorded the corneal curvature (Kmax), the central and thinnest corneal thicknesses, and the extent of protrusion of the front and back corneal surfaces. Another scan was used to measure the thickness of the tear film (moist layer), the outermost layer (epithelium), the Bowman layer, the main tissue (stroma), and the Descemet–endothelium part of the cornea.

Scleral thickness was measured from the limbus at 1 mm, 2 mm, and 3 mm in both nasal and temporal directions. Mean values were calculated from the AS-OCT images. Iris thickness was measured at 1 mm and 2 mm from the pupillary margin in both the nasal and temporal directions, and the mean values were used. In all cases, measurement points were standardized using anatomical landmarks to ensure repeatability and comparability.

To minimize the potential effects of pupillary dynamics on iris thickness measurements, all anterior segment optical coherence tomography examinations were performed under standardized ambient illumination conditions in a dimly lit room with controlled and constant background lighting, with patients allowed a brief adaptation period before image acquisition, while the device’s internal fixation target was consistently used and no pharmacologic agents affecting pupil size were administered, ensuring that all measurements were obtained under physiological, non-pharmacologically dilated conditions in accordance with previous studies emphasizing the importance of stable lighting for reliable iris morphometric assessment.

To further improve reproducibility, all measurements were acquired using the same AS-OCT device with identical imaging settings, including the scan protocol, focus, and magnification. Image acquisition was performed at similar times of day to minimize potential diurnal variations in pupil size and iris configuration. Only high-quality images with clearly delineated anterior and posterior iris borders were included in the analysis.

This standardized measurement protocol was applied consistently in both pre- and post-CXL examinations to ensure that observed changes in iris thickness reflected true structural alterations rather than variations in illumination or imaging parameters, as previously reported in anterior segment OCT studies. All measurements were performed by a single experienced examiner using the same standardized measurement protocol. The examiner was masked to the pre- and postoperative image status to minimize potential observer-related bias and variability.

### 2.4. Statistical Analysis

Statistical analyses were performed using IBM SPSS Statistics software (version 26.0; IBM Corp., Armonk, NY, USA). The distributional characteristics of continuous variables were assessed using visual methods (histograms and Q–Q plots) and the Shapiro–Wilk normality test. Continuous variables were expressed as mean ± standard deviation (mean ± SD).

Pre- and post-CXL measurements were compared using paired analyses, as the data represented matched observations from the same eyes. For variables showing a normal distribution, the paired-samples *t*-test was applied, whereas the Wilcoxon signed-rank test was used for variables that were not normally distributed. To quantitatively assess the magnitude of the treatment effect, effect sizes were calculated using Cohen’s *d* and classified as small (0.2), medium (0.5), or large (0.8). Effect sizes were reported to facilitate the interpretation of clinical relevance, independent of statistical significance.

Categorical variables were presented as frequencies and percentages (%). All statistical tests were two-tailed, and a *p*-value < 0.05 was considered statistically significant. To reduce the potential inflation of type I error associated with multiple comparisons, an additional false discovery rate (FDR) correction was performed using the Benjamini–Hochberg procedure. Adjusted significance levels were considered during the interpretation of the findings, together with effect sizes and clinical relevance. Given the evaluation of multiple structural parameters, the results were primarily interpreted in terms of effect sizes and clinical relevance.

ChatGPT (OpenAI, GPT-5.5) was used exclusively for language editing and improvement of manuscript readability. The required Artificial Intelligence Disclosure Statement has been added to the manuscript.

## 3. Results

A total of 94 eyes were included in the study, with a mean patient age of 26.56 ± 9.02 years. Findings obtained during the early postoperative follow-up period (3 months ± 2 weeks) are presented to characterize the structural changes observed after CXL. The mean follow-up duration was 5.2 ± 1.3 months.

**Figure 1:**
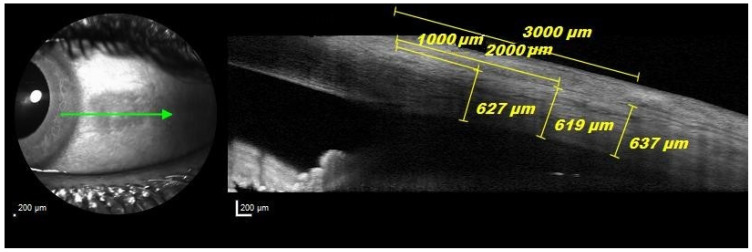
Measurement of scleral thickness using anterior segment optical coherence tomography (AS-OCT).

During the early postoperative follow-up period after CXL, opposing changes were observed in scleral and iris thickness. Scleral thickness, measured with the limbus as the reference point, showed a statistically significant increase at all measured distances compared with pre-CXL values; this increase was notably significant at the 1 mm and 2 mm measurement points, with medium effect sizes (*p* < 0.001) (Figure 1).

Representative anterior segment OCT image demonstrating scleral thickness measurements performed with reference to the limbus. Scleral thickness was measured at predefined distances of 1 mm, 2 mm, and 3 mm from the limbus in standardized AS-OCT cross-sectional images. Thickness values (µm) were obtained by measuring the perpendicular distance between the outer scleral boundary and the inner scleral interface. All measurements were performed using identical anatomical landmarks to ensure repeatability and comparability.

**Figure 2:**
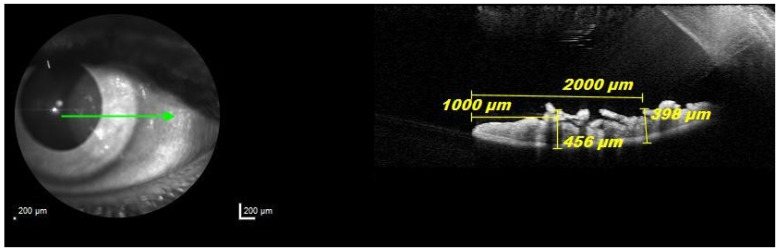
Measurement of iris thickness using anterior segment optical coherence tomography (AS-OCT).

Statistically significant but more modest increases were also observed at more peripheral measurement locations. In contrast, iris thickness decreased significantly at both recorded measurement points in the early postoperative period following CXL, with these reductions being associated with large effect sizes (*p* < 0.001) (Table 1, Figure 2).

Representative anterior segment OCT image illustrating iris thickness measurements performed with reference to the pupillary margin. Iris thickness was measured at standardized distances of 1 mm and 2 mm from the pupillary border on the nasal and temporal sides. Thickness values (µm) were obtained by measuring the perpendicular distance between the anterior and posterior borders of the iris stroma using standardized anatomical landmarks to ensure measurement repeatability and comparability.

**Figure 3:**
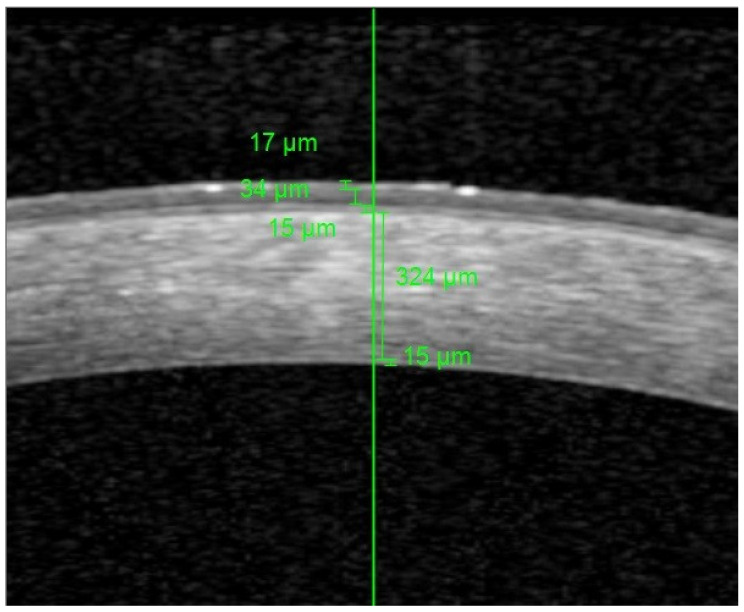
Measurement of corneal layer thicknesses using anterior segment optical coherence tomography (AS-OCT).

During the early postoperative follow-up period after CXL, selective and layer-specific changes were observed in tear film and corneal layer thicknesses. Tear film and corneal epithelial thicknesses showed significant reductions in the post-CXL period, with these changes associated with small to medium effect sizes (*p* < 0.05) (Figure 3). In contrast, no statistically significant differences were observed in the thickness of the Bowman layer or the Descemet–endothelial complex between the pre- and post-CXL periods. Stromal thickness demonstrated a statistically significant yet limited reduction during the early postoperative period following CXL (Table 2).

Representative anterior segment OCT image demonstrating corneal layer thickness measurements obtained from a central corneal cross-sectional scan. Thickness measurements of the tear film, epithelium, Bowman layer, stroma, and the Descemet–endothelium complex were performed at the corneal apex using perpendicular caliper placement between clearly defined anatomical boundaries. Centralized acquisition and standardized measurement protocols were applied to ensure repeatability and comparability of corneal layer assessments.

Evaluation of corneal topographic and tomographic parameters revealed heterogeneous, parameter-specific changes during the early postoperative period after CXL. In the anterior surface analysis, slight but statistically significant reductions were observed in anterior refractive power and mean keratometry, whereas anterior surface astigmatism and central elevation showed significant increases. On the posterior surface, posterior astigmatism and central elevation values increased significantly in the post-CXL period, while no significant changes were detected in the majority of posterior keratometric parameters. Pachymetric assessment showed significant thinning at the pupil center, corneal apex, and thinnest point during the early postoperative period after CXL; in contrast, Kmax and anterior chamber depth measurements remained comparable between the pre- and post-CXL periods (Table 3).

## 4. Discussion

In this study, eyes with progressive keratoconus that underwent CXL showed significant, opposing changes in corneal parameters and in scleral and iris thicknesses during the early postoperative period (1–3 months). Data from our study suggest that the early effects of CXL may not be confined solely to the cornea, as measurable structural responses in adjacent anterior segment tissues, including scleral and iris thickness alterations, provide relatively rare evidence supporting extra-corneal structural changes following CXL. Notably, the consistent increase in scleral thickness across all measurement points using the limbus as a reference, together with the marked and uniform decrease in pupil-referenced iris thickness, indicates that CXL may induce tissue-specific and heterogeneous responses within the anterior segment.

In recent years, the concept that keratoconus may not be a disease confined solely to the cornea but rather be part of a larger ocular biomechanical system has gained increasing support. This perspective is particularly relevant to comprehending the structural changes observed in extra-corneal anterior segment tissues during the early post-CXL period. Burguera-Giménez et al. demonstrated that the anterior scleral thickness profile differs between eyes with keratoconus and healthy controls, suggesting that the sclera may also contribute to the ectatic process [11]. In the present study, the post-CXL increase in scleral thickness supports the hypothesis that the biomechanical effects of treatment may not be limited to the corneal stroma but may extend to adjacent scleral tissues via the limbal region. Experimental and modeling-based studies have shown that biomechanical stiffening of corneal collagen fibers can be transmitted to the sclera through the limbal zone, leading to remodeling of scleral collagen organization [12,13,14,15,16]. This mechanism may provide a likely explanation for the observed increase in scleral thickness.

Complementing the scleral findings, the present study demonstrated a measurable and consistent reduction in measured iris thickness during the early postoperative period following CXL, indicating that dynamic anterior segment tissues may exhibit distinct structural responses to the procedure. The relatively large effect sizes observed for the reduction in iris thickness should be interpreted with caution. Although illumination conditions were standardized during AS-OCT acquisition, several physiological factors—including variations in tissue hydration, postoperative inflammatory responses, and autonomic regulation of the iris—may influence iris morphometry. Therefore, the observed decrease in iris thickness should not necessarily be interpreted as definitive structural atrophy but rather as a morphometric change observed in the early postoperative period.

In addition, postoperative organic responses may also contribute to the documented structural changes. Early postoperative inflammation, transient stromal edema, corneal haze formation, and the use of topical corticosteroids may influence anterior segment tissue hydration and hydrostatic balance [10,17,18]. Such factors could potentially contribute to the observed increase in scleral thickness and reduction in measured iris thickness during the early postoperative period.

Although previous studies have reported minimal changes in anterior chamber depth and pupillary dynamics after CXL, the number of studies directly and quantitatively evaluating changes in iris thickness remains limited [17,19,20]. The biomechanical and hydrostatic re-equilibration occurring within the anterior segment after CXL may result in structural redistribution or transient volume loss within the iris stroma. In addition, inflammatory mediators and local vascular responses in the early postoperative period have been suggested to contribute to morphological changes in anterior segment tissues [17,21,22,23]. Collectively, these findings support the concept that early postoperative responses after CXL may involve broader anterior segment structural adaptations [13,16,24].

In our study, evaluation of corneal topographic and tomographic parameters indicated no marked deterioration suggestive of keratoconus progression during the early postoperative period following CXL, while a significant keratometric improvement had not yet emerged. In particular, the absence of statistically significant changes in Kmax and mean keratometry values suggests that marked keratometric alterations were not observed during the early postoperative period. The results are consistent with previous reports indicating that the principal effect of CXL is stabilization, whereas topographic or keratometric improvements tend to become more apparent at later follow-up periods in most patients [18,25,26,27,28,29].

It should also be emphasized that the follow-up period of this study was limited to the early postoperative phase (approximately 3 months). Therefore, the structural changes observed in the cornea, sclera, and iris likely reflect early postoperative dynamics rather than long-term stabilization or remodeling processes after CXL. Previous studies have shown that some corneal and anterior segment changes following CXL may partially reverse or normalize over time [17,18,25]. Consequently, the present findings should not be extrapolated to long-term biomechanical behavior.

The principal contribution of this study lies in the simultaneous evaluation of thickness measurements of extra-corneal anterior segment tissues, such as the sclera and iris, together with corneal layer thicknesses and tomographic parameters during the early postoperative period following CXL. Because quantitative evidence that has quantitatively examined extra-corneal anterior segment tissues after CXL remains scarce, our data furnish complementary early-phase data to the existing literature.

The potential functional implications of the observed changes in scleral and iris thickness remain uncertain. Although the magnitude of the measured changes approached 50–60 µm in some regions, the clinical significance of such alterations is not yet clear. The magnitude of the observed scleral and iris thickness changes, approaching approximately 50–60 µm in some regions, exceeded the range of expected minor physiological fluctuations and is unlikely to be explained solely by measurement noise. All measurements were obtained using a standardized AS-OCT acquisition protocol, including identical imaging settings, controlled illumination conditions, anatomical landmark-based measurements, and assessment by a single experienced examiner to minimize variability and improve measurement repeatability. Nevertheless, physiological variability and device-related repeatability characteristics may partially contribute to the findings. From a biological perspective, postoperative inflammatory responses, transient tissue hydration alterations, and early structural remodeling processes may contribute to the magnitude of the observed changes during the postoperative period. Given the limited number of studies quantitatively evaluating scleral and iris thickness changes after CXL, direct comparisons with previous literature remain difficult. Therefore, these observations should be interpreted cautiously and require validation in future prospective studies. In theory, variations in iris morphology could influence pupillary dynamics or anterior chamber configuration, while changes in scleral structure could affect global ocular biomechanics. However, parameters such as pupillary function, anterior chamber volume, or intraocular pressure–related outcomes were not specifically evaluated in this study. Therefore, the functional consequences of these morphometric changes remain speculative and demand further investigation in future studies.

Nevertheless, several limitations should be considered when interpreting the present results. The retrospective design of the study and the relatively short early follow-up period of 1–3 months limit the assessment of the long-term course of the recorded structural alterations and their relationship with clinical outcomes. The absence of a healthy control group and the dependence on data from a single center represent additional limitations. Furthermore, the functional and clinical implications of the observed changes in scleral and iris thickness were not directly evaluated in this study. Additional correlation analyses evaluating the relationships between corneal structural changes and alterations in scleral or iris thickness were beyond the scope of the present study design and were not performed in the current analysis. However, investigating such associations may provide further mechanistic insight into anterior segment biomechanical interactions and improve understanding of tissue-specific responses following corneal collagen cross-linking. Future prospective studies incorporating correlation analyses may further strengthen the understanding of these structural relationships. Therefore, future prospective studies with longer follow-up periods and incorporating functional assessments are justified to better explain the clinical significance of this evidence. Accordingly, the structural changes observed in scleral and iris thickness should be interpreted as associative rather than direct causal consequences of CXL. These alterations may partly reflect transient postoperative inflammatory responses, changes in tissue hydration, or short-term physiological variability. Further investigations incorporating healthy control groups or untreated keratoconus eyes are warranted to clarify underlying mechanisms.

## 5. Conclusions

The findings of this study suggest that corneal collagen cross-linking (CXL) in eyes with progressive keratoconus is associated with measurable early structural changes in both corneal parameters, but also in extra-corneal anterior segment tissues, such as the sclera and iris, during the early postoperative period (3 months). The consistent increase in scleral thickness, accompanied by a reduction in measured iris thickness, suggests that structural alterations may be observed in multiple anterior segment tissues following CXL. Evaluation of corneal layer thicknesses together with topographic and tomographic parameters demonstrated that no statistically significant worsening was observed during the early postoperative period, although significant keratometric improvement had not yet emerged, supporting the existing literature that stabilization is the primary effect of CXL. Taken together, these results show that CXL should be considered not simply as a corneal intervention but as a procedure capable of influencing anterior segment biomechanics more broadly, and that extra-corneal anterior segment parameters may serve as complementary markers for tracking treatment response in future studies. The results should be interpreted in the context of early postoperative changes, and further studies with longer follow-up periods are required to determine whether these alterations persist or normalize over time.

## Figures and Tables

**Table 1:** Changes in Scleral and Iris Thickness After Corneal Cross-Linking in the Early Postoperative Period (*N* = 94).

Parameter	Pre-CXL (Mean ± SD)	Post-CXL (Mean ± SD)	*p* Value	Cohen’s *d*
Scleral thickness at 1 mm	567.55 ± 77.85.	628.59 ± 107.10	<0.001	0.65
Scleral thickness at 2 mm	563.36 ± 76.00	619.00 ± 103.04	<0.001	0.63
Scleral thickness at 3 mm	607.27 ± 85.83	629.63 ± 82.04	0.04	0.27
Iris thickness at 1 mm	478.38 ± 67.09	427.06 ± 55.45	<0.001	0.83
Iris thickness at 2 mm	466.33 ± 95.69	385.47 ± 52.72	<0.001	1.03

Abbreviations: CXL, corneal collagen cross-linking; SD, standard deviation; mm, millimeter.

**Table 2:** Changes in Tear Film and Corneal Layer Thicknesses After Corneal Cross-Linking (*N* = 94).

Parameter	Pre-CXL (Mean ± SD)	Post-CXL (Mean ± SD)	*p* Value	Cohen’s *d*
Tear film layer	23.73 ± 4.54	22.24 ± 4.25	0.02	0.34
Epithelium	29.55 ± 7.80	24.81 ± 8.67	<0.001	0.57
Bowman layer	15.73 ± 4.08	15.14 ± 5.55	0.33	0.12
Stroma	404.64 ± 38.71	395.36 ± 49.68	0.04	0.21
Descemet–endothelial complex	15.09 ± 3.30	15.14 ± 3.80	0.88	0.01

Abbreviations: CXL, corneal collagen cross-linking; SD, standard deviation.

**Table 3:** Changes in Corneal Topographic and Tomographic Parameters After Corneal Cross-Linking (*N* = 94).

Parameter	Pre-CXL (Mean ± SD)	Post-CXL (Mean ± SD)	*p* Value	Cohen’s *d*
Anterior K1	46.76 ± 5.96	45.10 ± 5.11	0.01	0.30
Anterior K2	51.23 ± 6.73	51.08 ± 5.55	0.68	0.02
Anterior Km	48.86 ± 6.28	47.85 ± 5.07	0.04	0.18
Anterior astigmatism	4.45 ± 2.09	5.96 ± 3.49	<0.001	0.51
Anterior Rmin	6.18 ± 1.00	5.98 ± 0.83	0.01	0.22
Anterior central elevation	23.59 ± 19.00	33.68 ± 26.50	<0.001	0.43
Posterior K1	−6.92 ± 0.95	−6.77 ± 0.92	0.02	0.16
Posterior K2	−7.84 ± 1.10	−7.85 ± 1.04	0.91	0.01
Posterior Km	−7.33 ± 1.00	−7.25 ± 0.95	0.31	0.08
Posterior astigmatism	0.92 ± 0.41	1.10 ± 0.45	<0.001	0.41
Posterior Rmin	4.41 ± 0.95	4.31 ± 0.69	0.04	0.12
Posterior central elevation	56.27 ± 35.37	68.95 ± 34.66	0.01	0.36
Pachymetry at pupil center	469.00 ± 43.13	444.91 ± 58.02	<0.001	0.46
Pachymetry at apex	462.82 ± 46.62	439.18 ± 68.77	<0.001	0.40
Thinnest pachymetry	452.45 ± 49.77	423.05 ± 59.02	<0.001	0.53
Kmax	55.91 ± 8.88	56.42 ± 7.76	0.32	0.06
Anterior chamber depth	3.35 ± 0.45	3.37 ± 0.34	0.54	0.05

Abbreviations: CXL, corneal collagen cross-linking; SD, standard deviation; K1, flat keratometry; K2, steep keratometry; Km, mean keratometry; Rmin, minimum radius of curvature; Kmax, maximum keratometry.

## Data Availability

The datasets generated and/or analyzed during the current study are available from the corresponding author upon reasonable request.
